# Association of Smoking with Chronic Kidney Disease Stages 3 to 5: A Mendelian Randomization Study

**DOI:** 10.34133/hds.0199

**Published:** 2024-11-04

**Authors:** Zhilong Zhang, Feifei Zhang, Xiaomeng Zhang, Lanlan Lu, Luxia Zhang

**Affiliations:** ^1^ Institute of Medical Technology, Peking University Health Science Center, Beijing, China.; ^2^ National Institute of Health Data Science at Peking University, Peking University Health Science Center, Beijing, China.; ^3^ State Key Laboratory of Vascular Homeostasis and Remodeling, Peking University, Beijing, China.; ^4^ Centre for Global Health, Usher Institute, University of Edinburgh, Edinburgh, UK.; ^5^ Xiaying Primary Health Care Center, Ningbo Yinzhou No.2 Hospital, Ningbo, Zhejiang Province, China.; ^6^ Renal Division, Department of Medicine, Peking University First Hospital, Peking University Institute of Nephrology, Beijing, China.; ^7^ Research Units of Diagnosis and Treatment of Immune-Mediated Kidney Diseases, Chinese Academy of Medical Sciences, Beijing, China.; ^8^ Advanced Institute of Information Technology, Peking University, Hangzhou, China.

## Abstract

**Background:** Previous studies suggested that smoking behavior (e.g., smoking status) was associated with an elevated risk of chronic kidney disease (CKD), yet whether this association is causal remains uncertain. **Methods:** We used data for half million participants aged 40 to 69 years from the UK Biobank cohort. In the traditional observational study, we used Cox proportional hazards models to calculate the associations between 2 smoking indices—smoking status and lifetime smoking index and incident CKD stages 3 to 5. Mendelian randomization (MR) approaches were used to estimate a potential causal effect. In one-sample MR, genetic variants associated with lifetime smoking index were used as instrument variables to examine the causal associations with CKD stages 3 to 5, among 344,255 UK Biobank participants with white British ancestry. We further validated our findings by a two-sample MR analysis using information from the Chronic Kidney Disease Genetics Consortium genome-wide association study. **Results:** In the traditional observational study, both smoking status [hazard ratio (HR): 1.26, 95% confidence interval (CI): 1.22 to 1.30] and lifetime smoking index (HR: 1.22, 95% CI: 1.20 to 1.24) were positively associated with a higher risk of incident CKD. However, both our one-sample and two-sample MR analyses showed no causal association between lifetime smoking index and CKD (all *P* > 0.05). The genetic instruments were validated by several statistical tests, and all sensitivity analyses showed similar results with the main model. **Conclusion:** Evidence from our analyses does not suggest a causal effect of smoking behavior on CKD risk. The positive association presented in the traditional observational study is possibly a result of confounding.

## Introduction

Approximately 10% of the global population are affected by chronic kidney disease (CKD), and this burden is escalating with the aging of the global population [1]. CKD is associated with increased risks of cardiovascular diseases and end-stage kidney disease, which could lead to reduced life expectancy and quality of life [2]. Currently, the therapeutic options for CKD are limited, with management primarily aimed at controlling diabetes, blood pressure, and complications such as cardiovascular diseases, anemia, and metabolic bone disease [3–5]. Therefore, the identification of the risk factors for CKD and the corresponding preventing strategies are essential to alleviate the disease burden.

Smoking behavior is a well-known risk factor for multiple morbidities [6,7]. Regarding its association with CKD, many studies have yielded inconclusive results [8–25]. For example, several observational studies suggested that current smoking status was associated with decreased kidney function and a high risk of CKD [10,12,13,26]. By contrast, results from some studies suggested that the association between smoking status and CKD incidence was not significant [17,20,27,28]. These contradictory findings may be due to confounding bias, reverse causation, and limited sample size in traditional observational studies [29]. For instance, an analysis using data from the Ongoing Telmisartan Alone and in Combination with Ramipril Global Endpoint Trial (ONTARGET) revealed that current smoker had a 19% increased risk of CKD progression, while analyses focusing on patients with diabetes showed no such association. This finding suggested that diabetes might be a potential confounder for the association between smoking and CKD progression [30].

Mendelian randomization (MR), as an instrumental variable approach, can overcome the limitations from traditional observational studies and provide new insights into the potential causal relationship between exposure and outcome [31,32]. Using genetic variants as instrumental variables, this approach has been widely applied in recent studies and has provided valuable insights for the complex relationships between various life style factors and diseases [1,6,33]. In this study, we aimed to evaluate whether smoking behavior was causally associated with CKD via MR analyses. We performed a traditional observational study and MR analyses of smoking and CKD using both individual-level data in UK Biobank (UKB) and published summary-level statistic data.

## Methods

### Methods overview

In this study, we first performed a traditional observational study to assess the statistical association between smoking and CKD based on the UKB data. Based on the same dataset, we then conducted a one-sample MR analysis to evaluate the potential causal association between smoking and CKD. One-sample MR is free from the issue of sample overlap and can perform detailed analysis of interactions, for example, nonlinear and subgroup analyses, but it is prone to data overfitting [34]. Therefore, we additionally performed a two-sample MR by using summary-level statistics. One-sample MR and two-sample MR compensate each other, and combining the results from both methods can provide a more valid and comprehensive evaluation of causal associations. The results of MR analyses are reported according to STROBE-MR statement [35].

### Study population

This study utilized individual-level data from the UKB for the traditional observational study and one-sample MR. The UKB cohort included >500,000 participants aged 40 to 69 years in the United Kingdom, recruited from 2006 to 2010 in 22 assessment centers [36]. All participants provided informed consent and underwent clinical interviews, anthropometric assessments, and laboratory tests. In UKB, we defined the outcome as the development of CKD stages 3 to 5. We treated the International Classification of Diseases 10th Revision (ICD-10) codes N18.0, N18.3, N18.4, N18.5, N18.8, N18.9, I12.0, I13.1, and I13.2 and ICD-9 code 585.9 as indicative of CKD. An additional report was also used for algorithmically defined end-stage renal diseases (ESRD). Detailed information on the process of participant filtration are shown in Supplementary Methods.

### Smoking index

In the traditional observational study, we generated 2 smoking indices including smoking status (never or ever) and the lifetime smoking index. The lifetime smoking index was generated by Wootton et al. [37] and included information about smoking intensity, duration, and smoking initiation and cessation. The lifetime smoking index for each participant is defined as

$$I=\left(1-0.5^{\frac{\mathrm{dur}}{18}}\;\right)0.5^{\frac{\mathrm{tsc}}{18}}\log\left(\mathrm{int}+1\right),$$ (1)

where “dur” denotes the time of smoking duration in years, “tsc” denotes the time since cession in years, and “int” denotes the average cigarettes smoked per day. Based on the definition, the index of an individual who smoked 20 cigarettes daily for 15 years and quit 17 years ago is equal to the index of an individual who smoked 60 cigarettes daily for 13 years and quit 22 years ago [37]. The index in UKB data was calculated from smoking status (field ID 2011), age at initiation in years (field IDs 3436 and 2867), age at cessation in years (field ID 2897), and number of cigarettes smoked per day (field ID 3456/2887).

In both one-sample and two-sample MR analyses, we only used the lifetime smoking index as the exposure. As mentioned earlier, the lifetime smoking index is a continuous exposure variable that includes more aspects of smoking behavior such as smoking intensity, smoking initiation and duration, and smoking cessation. By contrast, the smoking status is a binary exposure variable that can be seen as a simplified version of lifetime smoking index, i.e., smoking status equals 1 when the smoking index is positive, and smoking status equals 0 when the smoking index is 0. Furthermore, smoking status as a dichotomized exposure may complicate the interpretation of causal estimates and not fully capture the causal relationship [38,39].

### Genetic instruments

In one-sample MR analysis, single-nucleotide polymorphisms (SNPs) were selected based on 2 criteria. First, the SNPs should be associated with the lifetime smoking index at a genome-wide significance threshold (*P* < 5 × 10^−8^), based on the results in a latest published genome-wide association studies (GWASs) [37]. Second, the SNPs should be directly measured and reported by the UKB. We did not use imputed SNPs to avoid potential confounding [40]. Under these 2 criteria, 14 SNPs were selected (Table S1) and they were further combined into a polygenic risk score (PRS) and used as a genetic instrument to avoid weak instrument bias [41]. The PRS for each individual was calculated by summing the count of risk alleles for the SNPs, weighted by the effect size of these SNPs on lifetime smoking index based on published results [37]. The PRS for a participant can be expressed as:

$$P=\sum_{i=1}^{M}{\mathrm{SNP}}_{i}*\beta_{i},$$ (2)

where *SNP_i_* is the count of risk alleles (0, 1, or 2) carried at the *i*th SNP, *β_i_* is the effect size of this SNP on lifetime smoking index, and *M* is the total number of selected SNPs [41].

For two-sample MR analysis, we examined 42 SNPs strongly associated with lifetime smoking index identified in a GWAS study of 462,690 individuals of European ancestry from UKB cohort (Table S2) [37]. The summary-level data for CKD were extracted from a published study performed by the Chronic Kidney Disease Genetics Consortium (CKDGen) [42]. In this study, incident CKD was defined as estimated glomerular filtration rate (eGFR) < 60 ml/min/1.73 m^2^, and a total of 12,385 cases and 104,780 controls were included. Previous study suggested that genetic instruments for eGFR have sufficient power to capture the risk of various kidney diseases including CKD [43].

### Statistical methods

#### Traditional observational study

Hazard ratios (HRs) and 95% confidence intervals (CIs) for CKD risk in relation to the smoking status and lifetime smoking index were calculated by fitting Cox proportional hazards models. Both unadjusted and adjusted models were considered. To explore the potential nonlinear exposure–response relationships, we fitted a penalty spline with 2 degrees of freedom (df) to the lifetime smoking index [44]. Penalty splines with df of 3 and 4 were also fitted as sensitivity analyses. The covariates used for the adjusted model included sex, age, assessment center, ethnicity, annual household income, current employment status, qualifications, activity intensity, body mass index (BMI), alcohol intake intensity, diet quality, sleep duration, and comorbidities (diabetes, hypertension, dyslipidemia, and nonalcoholic fatty liver disease) at baseline [45]. Details on the covariates are shown in Supplementary Methods.

#### One-sample MR analysis

We used a 2-stage least squares (2SLS) method to evaluate the causal association between the lifetime smoking index and CKD based on individual-level data from UKB. In the first stage, the lifetime smoking index was regressed on the PRS. Next, logistic regression analysis was conducted and the odds ratio (OR) and 95% CI of CKD were estimated based on the fitted values of smoking index from the first-stage analysis. Age, sex, assessment centers, and top 10 genetic principal components were adjusted in both stages. We adjusted a smaller number of covariates in MR analyses than in the traditional observational study because in such analysis using genetics as instrumental variables, there is no need to adjust for many covariates that might otherwise bias the causal estimates [39]. To alleviate the noncollapsibility issue in the second-stage logistic regression model, we used G-estimation method for unbiased estimation of the causal OR values [46,47]. As the observational study, we also performed nonlinear MR using localized average causal effect (LACE) to evaluate the nonlinear association between genetic predicted lifetime smoking index and CKD [48]. Specifically, we stratified the population into 3 clusters based on the distribution of the residual of lifetime smoking index regressed on the PRS. Then, we performed the piecewise linear MR analysis and calculated the *P* values from 2 tests for nonlinearity—the quadratic test and Cochran’s *Q* test [48].

Subgroup analyses were performed to account for possible differences in genetic architecture [49]. To avoid collider bias [50], we performed subgroups analyses by age, sex, and covariates for which the genetic instruments have been validated, including hypertension, diabetes, and BMI (Table S3). The definition of hypertension and diabetes was the same as the observational study (Supplementary Methods).

We performed several sensitivity analyses to validate the results of one-sample MR. First, to avoid confounding caused by pleiotropy, we recalculated the PRS by removing SNPs that showed genome-wide significant association with key potential confounders, such as hypertension, diabetes, and BMI (Table S3). Second, to show the direct effects of smoking, we directly adjusted for the 3 confounders including BMI, diabetes, and hypertension in the model and excluded SNPs associated with the 3 confounders. Finally, to further alleviate the impact of potentially invalid SNPs, we performed a leave-one-out analysis by excluding each SNP one at a time when constructing PRS. We used an online tool to calculate the statistical power of the MR analyses [51].

#### Two-sample MR analysis

As in one-sample MR analysis, we included SNPs that were genome-wide significantly associated with the exposure (*P* < 5 × 10^−8^). To prevent linkage disequilibrium (LD) and correlated instrument variables, we clumped the SNPs to kb > 10,000 and *r*^2^ < 0.0001 using “clump_data” function from “TwoSampleMR” R package [38]. We performed 3 statistical tests for the validity of the selected SNPs. Heterogeneity refers to the variability in the association between the exposure variable and the outcome variable across various genetic variants, and the existence of heterogeneity across SNPs may cause an invalid MR result. We tested the selected SNPs using Cochran’s *Q* test for heterogeneity [52]. Horizontal pleiotropy occurs when a genetic instrument influences multiple phenotypes, potentially violating the exclusion restriction assumption of genetic instrument. We used the MR-PRESSO global test [53] and the MR-Egger intercept test [54] to assess the potential pleiotropy.

We used 5 different MR methods with different underlying assumptions: (a) The conventional fixed effect inverse variance method was the main method for two-sample MR, which uses inverse-weighted meta-analysis to combine the estimates of causal effect with different genetic instruments [1]. (b) The maximum likelihood method was also used to calculate causal estimates [55]. (c) To avoid potential bias from directional pleiotropy, MR-Egger regression was performed as a sensitivity analysis [54]. MR-Egger can estimate the pleiotropy parameter as the intercept of the model and yield robust causal estimates. Furthermore, (d) the weighted median method and (e) the weighted mode method were implemented as additional sensitivity analyses [56]. For an unbiased estimation, the weighted median and weighted mode method require that at least 50% and the majority of the genetic instruments are valid, respectively.

All statistical analyses were conducted using R software (R Foundation, Vienna, Austria). Package “survival” was used to conduct observational study; “ivtools” was used to conduct one-sample MR analysis; “TwoSampleMR” was used to conduct two-sample MR analysis.

## Results

### Study population characteristics in UKB

**Fig. 1.**
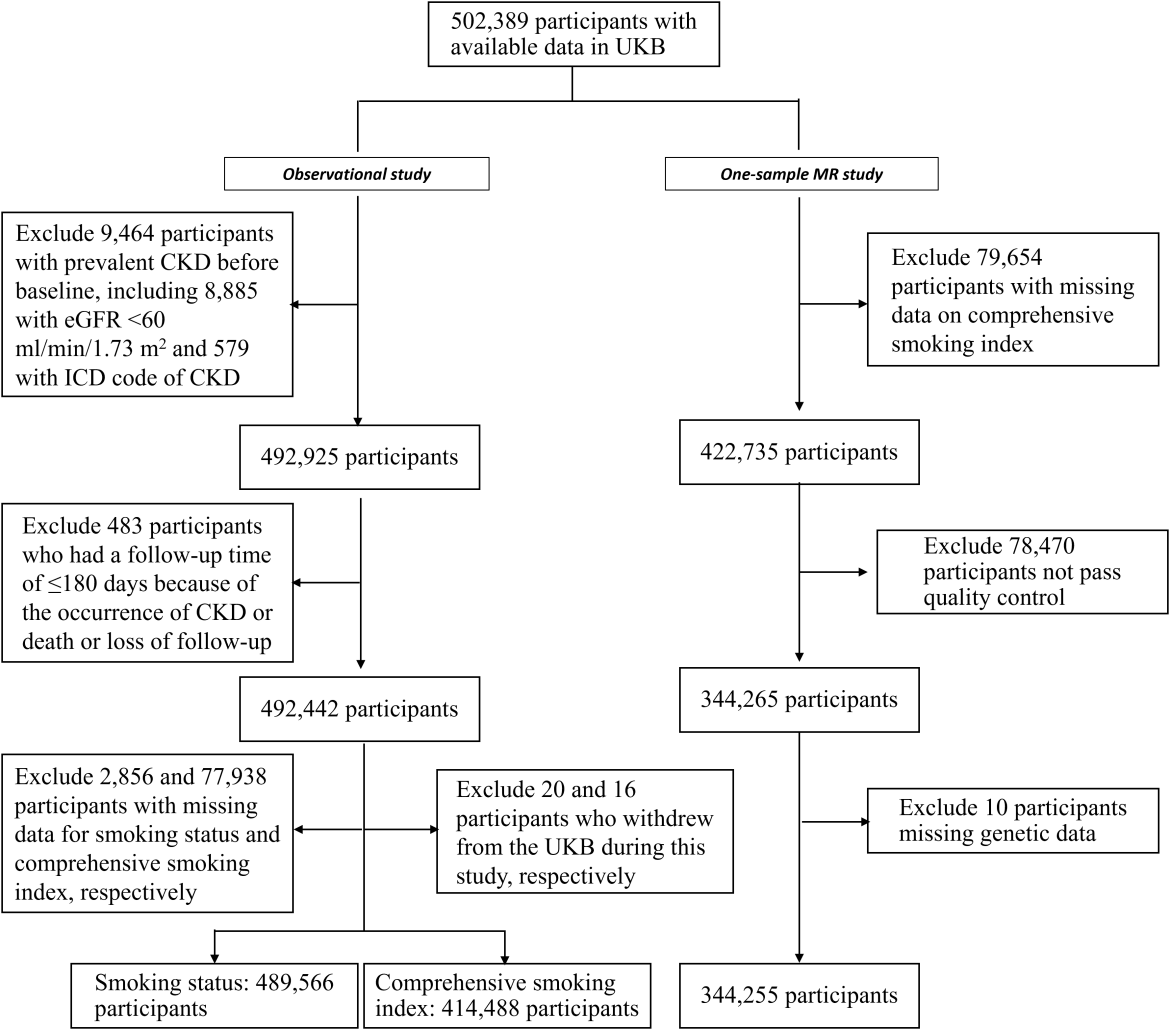
Flowchart for participant selection

A total of 489,566 and 414,488 participants were included in the traditional observational study for smoking status and lifetime smoking index, respectively (Fig. 1). In the traditional observational study, 90,742 participants were excluded due to either preexisting CKD diagnosis before the baseline, follow-up period <180 d, or missing smoking status/lifetime smoking index (Fig. 1). Of the 489,566 participants included in the traditional observational study, 15,135 (3.1%) participants developed CKD over a median 12.8 years (4,675 d/365.25) of follow-up (Table 1). A total of 220,752 (45.1%) participants were reported to have ever smoked. Compared to nonsmokers, smokers were more likely to develop CKD, to be unemployed, to not have a college/university degree, to have a lower income, and to have a BMI of ≥30 kg/m^2^ (Table 1).

**Table 1.** Characteristics of the included participants stratified by smoking status

Variables	Total participants (*n* = 489,566)	Nonsmokers (*n* = 268,814)	Smokers (*n* = 220,752)
CKD, yes, *n* (%)	15,135 (3.1)	6,896 (2.6)	8,239 (3.7)
Age, years, median (IQR)	58.00 [50.00, 64.00]	57.00 [50.00, 63.00]	59.00 [52.00, 64.00]
Follow-up time, days, median (IQR)	4,675.00 [4,397.00, 4,942.00]	4,685.00 [4,417.00, 4,949.00]	4,663.00 [4,355.00, 4,932.00]
Sex, male, *n* (%)	222,693 (45.5)	109,510 (40.7)	113,183 (51.3)
Ethnicity, *n* (%)			
Non-white	26,019 (5.3)	18,056 (6.7)	7,963 (3.6)
White	461,852 (94.3)	249,883 (93.0)	211,969 (96.0)
Missing	1,695 (0.3)	875 (0.3)	820 (0.4)
Household income, *n* (%)			
<£18,000	93,510 (19.1)	44,425 (16.5)	49,085 (22.2)
≥£18,000	322,630 (65.9)	182,694 (68.0)	139,936 (63.4)
Missing	73,426 (15.0)	41,695 (15.5)	317,331(14.4)
Qualifications, *n* (%)			
College/university degree	158,577 (32.4)	96,540 (35.9)	62,037 (28.1)
Others	322,162 (65.8)	167,520 (62.3)	154,642 (70.1)
Missing	8,827 (1.8)	4,754 (1.8)	4,073 (1.8)
Current employment status, *n* (%)			
Non-employed	204,407 (41.8)	104,964 (39.0)	99,443 (45.0)
Employed	283,268 (57.9)	162,735 (60.5)	120,533 (54.6)
Missing	1,891 (0.4)	1,115 (0.4)	776 (0.4)
Activity intensity, *n* (%)			
Moderate/vigorous activity	298,569 (61.0)	164,580 (61.2)	133,989 (60.7)
Others	151,347 (30.9)	83,183 (30.9)	68,164 (30.9)
Missing	39,650 (8.1)	21,051 (7.8)	18,599 (8.4)
BMI, *n* (%)			
<30	369,345 (75.4)	207,149 (77.1)	162,196 (73.5)
≥30	117,651 (24.0)	60,306 (22.4)	57,345 (26.0)
Missing	2,570 (0.5)	1,359 (0.5)	1,211 (0.5)
Lifetime smoking index, median (IQR)	0.00 [0.00, 0.53]	0.00 [0.00, 0.00]	1.07 [0.46,1.83]
Alcohol intake, *n* (%)			
Nondrinkers	84,730 (17.3)	52,910 (19.7)	31,820 (14.4)
Normal drinkers	233,622 (47.7)	138,780 (51.6)	94,842 (43.0)
Heavy drinkers	135,226 (27.6)	55,314 (20.6)	79,912 (36.2)
Missing	35,988 (7.4)	21,810 (8.1)	14,178 (6.4)
Diet quality, good, *n* (%)	358,289 (73.2)	201,038 (74.8)	157,251 (71.2)
Sleep duration, *n* (%)			
Short	120,216 (24.6)	63,493 (23.6)	56,723 (25.7)
Intermediate	329,599 (67.3)	184,926 (68.8)	144,673 (65.5)
Long	36,772 (7.5)	18,735 (7.0)	18,037 (8.2)
Missing	2,979 (0.6)	1,660 (0.6)	1,319 (0.6)
Hypertension at baseline, yes, *n* (%)	127,499 (26.0)	64,091 (23.8)	63,408 (28.7)
Diabetes at baseline, yes, *n* (%)	24,105 (4.9)	11,055 (4.1)	13,050 (5.9)
Dyslipidemia at baseline, *n* (%)	71,081 (14.5)	32,920 (12.2)	38,161 (17.3)
NAFLD at baseline, *n* (%)	3,513 (0.7)	1,700 (0.6)	1,813 (0.8)

CKD, chronic kidney disease; IQR, interquartile range; BMI, body mass index; NAFLD, nonalcoholic fatty liver disease

In one-sample MR, 344,255 individuals of white British ancestry remained after excluding those lacking smoking data (79,654), who did not pass the quality control process (78,470), and missing genetic data (10; Fig. 1). Among the included participants, 13,573 (3.9%) participants were diagnosed with incident CKD.

### Traditional observational study

**Fig. 2.**
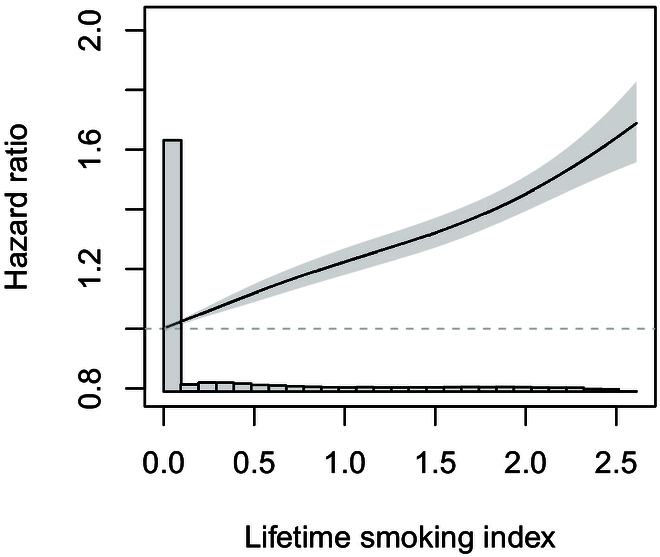
Exposure–response associations between lifetime smoking index and incident CKD. Data were trimmed from 1% to 99% percentiles of lifetime smoking index, and the 1% percentile of lifetime smoking index was used as the reference. The solid line represents the HRs, and the shaded area represents their 95% CIs. The histogram represents the distribution of lifetime smoking index.

In Cox proportional hazards models, we observed positive associations of both smoking status and the lifetime smoking index with CKD in both the unadjusted and the adjusted models (Table 2). Compared with nonsmokers, smokers had a high risk of developing CKD (HR: 1.26, 95% CI: 1.22 to 1.30). For per-unit increase in lifetime smoking index, the HR of CKD was 1.22 (95% CI: 1.20 to 1.24) in the adjusted model (Table 2). Lifetime smoking index was near-linearly associated with incident CKD (Fig. 2). Penalty splines with df of 3 and 4 showed similar nonlinear associations.

**Table 2.** Relationships between smoking indices and incident chronic kidney disease

Smoking indices	Unadjusted model		Adjusted model	
	HR (95% CI)	*P*	HR (95% CI)	*P*
Smoking status				
Never	1 (ref)	NA	1 (ref)	NA
Ever	1.50 (1.45–1.55)	<0.001	1.26 (1.22–1.30)	<0.001
Lifetime smoking index				
Every unit increase	1.39 (1.36–1.41)	<0.001	1.22 (1.20–1.24)	<0.001

HR, hazard ratio; CI, confidence interval

### One-sample MR analysis

The PRS was significantly associated with the lifetime smoking index (*P* < 2 × 10^−16^), and the *F* statistic was 310.46. The genetically predicted smoking index was not significantly associated with CKD outcome (2SLS: OR = 0.98, 95% CI: 0.44 to 2.17, *P* = 0.95; G-estimation: OR = 0.99, 95% CI: 0.49 to 2.00; Table 3). In addition, no significant nonlinearity was found between genetically predicted smoking index and incident CKD (Fig. S1). Subgroup analyses (Table 3) suggested similar nonsignificant associations among all subgroups as our main model.

**Table 3.** Risk of chronic kidney disease in overall participants and according to age, sex, diabetes, hypertension, and BMI for lifetime smoking index based on one-sample mendelian randomization analysis. Polygenic risk score (PRS) for lifetime smoking index was used as the genetic instrument.

Methods	OR (95% CI)	*P*
Overall population		
2SLS ^a^	0.98 (0.44–2.17)	0.95
G-estimation ^b^	0.99 (0.49–2.00)	0.99
Age ^c^		
<58	1.73 (0.07–41.45)	0.74
≥58	3.27 (0.71–15.09)	0.13
Sex		
Male	1.16 (0.31–4.24)	0.82
Female	0.86 (0.31–2.39)	0.77
Diabetes		
Diabetes	1.57 (0.10–24.54)	0.75
No-Diabetes	0.33 (0.03–3.08)	0.33
Hypertension		
Hypertension	2.04 (0.01–2.82)	0.24
No-Hypertension	1.50 (0.08–2.73)	0.78
BMI ^d^		
<25	0.04 (0.01–18.12)	0.30
≥25	0.64 (0.09–4.37)	0.65

OR, odds ratio; CI, confidence interval; 2SLS, 2-stage least squares

^a^ Main model using the 2-stage method, with age, sex, assessment centers, and the first 10 principal components of the genetic information adjusted.

^b^ G-estimation method was used for an unbiased estimation of the causal OR values when noncollapsibility issue exists in the nonlinear effect measure.

^c^ The median age of the included participants is 58.

^d^ The cutoff value for BMI is chosen as the threshold between normal weight and overweight, i.e., BMI < 25 and BMI ≥ 25.

Three SNPs (rs2062882, rs4949465, and rs6962772) were found to be associated with at least one of the considered confounders (BMI, hypertension, and diabetes; Table S3). Among them, one SNP (rs2062882) was directly associated with CKD outcome—reaching the Bonferroni-adjusted *P* value (<0.05/14 SNPs related to smoking index). Using newly calculated PRS by removing these SNPs has generated consistent results with the main model (Table S4). Similarly, the results of leave-one-out analysis are in line with the main analysis (Table S5). Based on the statistics derived from our one-sample MR results, our MR analyses had 99% power to detect a statistically significant causal association (2SLS: effect size = −0.02, 95% CI: −0.82 to 0.77) at a type-1 error rate of 5%.

### Two-sample MR analysis

**Fig. 3.**
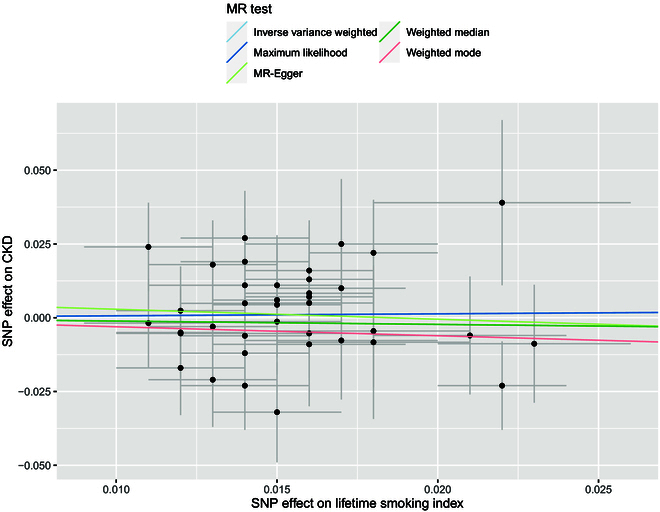
Scatterplot showing causal association of lifetime smoking index with the risk of CKD across 42 SNPs based on five two-sample MR methods. Slope of the colored lines indicates the magnitude of the causal associations.

We utilized 42 SNPs associated with smoking index from the GWAS study [37] for our MR analyses (Table S2). When testing for a causal effect of smoking index on CKD, there was no significant heterogeneity (*P* = 0.73 for heterogeneity test), and there was no significant pleiotropy (*P* = 0.68 for MR-Egger intercept test and *P* = 0.73 for MR-PRESSO global test). The 5 selected methods, i.e., inverse variance weighted method, maximum likelihood method, MR-Egger, weighted mean, and weighted mode, consistently suggested that there was no causal relationship between lifetime smoking index and CKD (Table 4 and Fig. 3).

**Table 4.** Risk of chronic kidney disease associated with lifetime smoking index based on the two-sample mendelian randomization analysis

	OR (95% CI)	*P*
Inverse variance weighted	1.07 (0.75–1.52)	0.715
Maximum likelihood	1.07 (0.75–1.52)	0.711
MR-Egger	0.71 (0.10–4.92)	0.734
Weighted median	0.89 (0.53–1.49)	0.668
Weighted mode	0.74 (0.23–2.33)	0.607

OR, odds ratio; CI, confidence interval

## Discussion

In this study, we estimated the potential causal association between smoking behavior and the development of CKD. In the traditional observational study, both smoking status and lifetime smoking index were associated with an elevated incidence of CKD. However, in the MR analysis, we found no evidence of a causal role of smoking behavior in incident CKD.

Findings from our observational study are in line with several previous studies [57,58] that smoking behaviors were strongly associated with incident CKD. Further, a recent meta-analysis [58] reported that current and previous smokers had significantly higher risk of CKD compared to never smokers. However, relationships between smoking and CKD in observational studies have been inconclusive [17,27,28]. For example, findings from a recent longitudinal cohort study with the Chronic Renal Insufficiency Cohort indicated no significant relationship between self-reported tobacco use and the progression of CKD [27].

Our MR analyses, however, have yielded insignificant relationships between CKD and smoking. Our findings support that smoking behavior may not have a total causal effect on CKD; the statistical associations between smoking and CKD are possibly the consequence of unmeasured confounding. As of 2023 July 26, we only found one previous study that has studied the causal relationship between smoking behavior and CKD using two-sample MR method and reported weak evidence of causal effect [59]. Compared with this study, our study has several advantages. First, our study has a larger power (>0.99 versus 0.387 in the previous study) to assess a causal relationship between smoking and CKD. Second, we employed strict selection criteria for genetic instruments and performed various statistical tests to assess the validity of the selected instrument. The instrument passed all statistical tests, and sensitivity analyses produced consistent results. By contrast, the results in the previous study varied by different estimation methods using the same UKB data. Third, one limitation of the previous study is that using only two-sample MR was unable to account for the sample overlap between the exposure and outcome GWAS datasets. The one-sample analysis supplemented in our study is free from the issue of sample overlap and thus avoids potentially biased estimates caused by overlapping datasets. Finally, our study performed analyses for a comprehensive evaluation of the potential causal relationship between smoking and CKD, including subgroup analyses and nonlinear analysis, and further ensured the robustness of our findings.

However, it is important to consider that the absence of a total causal effect does not preclude the possibility of both direct and indirect effects of smoking on CKD through various mediators. In the presence of mediators, the estimates of MR analysis are the combination of direct effect and indirect effect [60,61]. Previous observational studies have found that several covariates such as diabetes may confound the relationship between smoking and CKD. For example, a study leveraging data from ONTARGET found that current tobacco use was associated with 19% increased risk of CKD progression. However, when the analysis was confined to participants with diabetes, this association was not observed [30]. This insignificant finding may be due to the potential mediating influence of diabetes, which could obscure the direct effect of smoking on CKD progression. Other potential mediators include preexisting CKD [62] and hypertension [63]. In addition, previous MR studies have identified hypertension and diabetes as having causal effect on CKD [59,64]. In our MR analysis, we performed subgroup analyses according to the presence of hypertension and diabetes. However, the results from all subgroup analyses were not statistically significant across all strata. Subgroup analyses are prone to collider bias [50], and mediation MR is needed for unbiased estimation of mediation effect [65]. Given the substantial evidence linking smoking to CKD, smoking behavior should be considered as a risk factor for CKD through potential mediation mechanism, and a comprehensive mediation analysis is needed to elucidate the intricate interplay between various factors.

A valid instrumental variable when testing a causal relationship between modifiable factors and complex diseases using MR approach need to fulfill 3 core assumptions: (a) the relevance assumption: the genetic instrument and the assessed exposure should have strong association, (b) the exclusion restriction assumption: the genetic instrument should affect the risk of disease only through the assessed exposure, and (c) the independence assumption: the genetic instrument should be independent of confounders and pleiotropies [31]. Our study met these 3 assumptions. First, the genetic instruments created in this study are significantly associated with the smoking behavior phenotypes, so the relevance assumption is met. Second, although the exclusion restriction assumption cannot be directly tested, we assessed the validity of the instrument with sensitivity analyses, including removing potentially invalid SNPs, leave-one-out analysis, and adjusting the confounders. Third, our two-sample MR results of MR-PRESSO test and MR-Egger test showed that there was no significant pleiotropy. Furthermore, MR estimation methods that are robust to invalid SNPs have shown consistent negative results. Thus, the independence assumption would have been attained [31].

Our study has several limitations. First, the MR analysis was limited to individuals of European descendant, so the findings in the current study may not be generalizable to populations of other ethnical groups. Besides, the UKB recruited general population aged 40 to 69 years at recruitment, and this may limit the generalizability of conclusions from the study to population with underlying conditions. Second, the lifetime smoking index captures multiple aspects of smoking behavior, but as a composite index, its use as an instrument may introduce biases for horizontal pleiotropy. We conducted several sensitivity analyses to guarantee the robustness of our results. Furthermore, the MR-PRESSO test and MR-Egger intercepts did not show evidence of directional pleiotropy. Third, the lifetime smoking index has been collected via questionnaires, which may introduce recall bias and therefore lead to a biased risk estimate. Fourth, the genetic instrument in one-sample MR study was constructed from only 14 SNPs, which may result in weak instrument bias [66]. However, it has been demonstrated that the direction of the potential weak instruments bias in one-sample analysis should be aligned with the observational study, rather than the null hypothesis generated in the current study [67]. Furthermore, a higher *F* statistic (i.e., >10) of genetic instrument suggests smaller bias [67,68], and the *F* statistic in the present study was 310.46 for the PRS. Therefore, the bias, if there were any, shall be very limited. Finally, there may be an overlap of participants between the exposure and outcome datasets, which could cause bias in the estimates. However, this would only affect our study by producing a false positive result, but not a negative result [67].

## Conclusion

In conclusion, our MR analyses do not suggest a causal effect of smoking behavior on CKD, although our traditional observational study showed a positive correlation between them. This discrepancy, along with findings from previous researches, collectively implies that while smoking may not have a total causal effect on CKD, covariates such as diabetes and hypertension could be important mediators that have confounded observational analyses. Given the substantial evidence linking smoking to CKD, further mediation analysis is needed to understand the intricate mechanisms between smoking behavior and CKD. Such an analysis is essential for generating valuable insights that can inform and enhance public health strategies focused on mitigating CKD risk factors and improving overall kidney health.

## Data Availability

The genetic association data for one-sample and two-sample MR are available in the Supplementary Materials (Tables S1 and S2). The GWAS summary data used in this study are publicly available via the IEU OpenGWAS database (https://gwas.mrcieu.ac.uk/). Individual data from the UKB could be obtained upon request to the board.

## Supplementary Materials

Supplementary 1 Supplementary Methods Supplementary Results Fig. S1 Tables S1 to S5 References [69,70]
