# Supplementary material for: Association of Smoking with Chronic Kidney Disease Stages 3 to 5: A Mendelian Randomization Study
Source: Health Data Sci. 2024 Nov 4;4:0199. doi: 10.34133/hds.0199 (PMC11532587; doi:10.34133/hds.0199)
Supplement: Supplementary 1 — Supplementary Methods Supplementary Results Fig. S1 Tables S1 to S5 References [69,70] [file hds.0199.f1.zip › HDS-D-24-00012.pdf]

Supplementary Material for

Association of smoking with chronic kidney disease stages 3-5: A mendelian randomization study

Supplementary Methods

Study population

In the one-sample MR analysis, we obtained participants of European ancestry in UKB passing the individual-level quality control filter. Participants with excessive/minimal heterozygosity (Field ID 22027), sex mismatch (Field ID 22001), individuals with sex chromosome aneuploidy (Field ID 22019), non-European ancestry (Field ID 22006), excessive genetic relatedness (more than 10 putative third-degree relatives in the kinship table) or missing quality control data were excluded from the analysis.

In the observational study, participants with pre-existing CKD (indicated by ICD codes for CKD and/or estimated glomerular filtration rate <60 ml/min/1.73 m^2 and/or urinary albumin creatinine ratio > 300 mg/g) at the time of visiting assessment centers were excluded. Additionally, participants with a follow-up period less than 180 days were excluded to ensure adequate time for outcome to occur. The follow-up period encompassed the time from visiting assessment centers to the onset date of CKD or dates of other events including death, loss to follow-up, or the end of follow-up (31 December 2021). Participants who were lost to follow-up or died before CKD occurred were censored at the time of the respective event.

Covariates

Covariates which could potentially affect CKD onset were collected, including sex, age, assessment center, ethnicity (white or non-white), current employment status (employed or not), annual household income (< £18000 or ≥ £18000), qualifications (college/university or others), activity (moderate/vigorous activity or others), body mass index (BMI, <30 or ≥ 30 kg/m2),

alcohol intake intensity (none, normal or heavy), diet quality, sleep duration, and comorbidities (hypertension, diabetes, dyslipidemia, and non-alcoholic fatty liver disease) at baseline [45]. All the information was extracted from central registry/the touchscreen questionnaire or trained nurses measured them at the start.

Moderate/vigorous activity meant a participant did moderate/vigorous activities for over 10 minutes non-stop at a time in more than 4 days/week. BMI was weight (in kilograms) divided by height (in meters) squared. Alcohol intake intensity is a composite index reflecting the weekly units of alcohol intake (one unit = 10 ml of pure ethanol) across different categories [69]. When reported monthly, the intake was converted to units per week by dividing by 4.3. Alcohol categories reported by the participant include red wine, champagne/white wine, beer/cider, spirits, fortified wine, and other. We followed one previous study [69] to define the units of alcohol intake: 1) a pint or can of beer/lager/cider = two units; 2) a 25 ml single shot of spirits = one unit; and 3) a standard glass of wine = two units. Based on the index, we grouped participants into non-drinkers (consuming 0 unit/week), normal drinkers (i.e., women who reported consuming < 18 units/week and men who consumed < 24 units/week), and heavy drinkers (i.e., women who reported consuming >= 18 units/week and men who consumed >= 24 units/week). Diet quality is a composite index reflecting whether participants follow healthy diet patterns. We defined the healthy food items by following one previous study [70]: 1) Total fruit and vegetable intake > 4.5 pieces or servings a week. 2) Total fish intake > 2 per week. 3) intake of processed meat ≤ 2 times per week and intake of red meat ≤ 5 times per week. Diet quality was calculated by summing the total items of healthy food (0, 1, 2, 3).

Comorbidities were extracted using the ICD-10 codes from the first occurrences data, and treated as binary variables (yes or no), including hypertension (I10, I11, I12, I13 and I15), diabetes (E10, E11, E12, E13 and E14), Dyslipidemia (E78) and NAFLD (K75 and K76).

Supplementary Results

Table S1 Single-nucleotide polymorphisms and effect sizes for lifetime smoking index identified in UK Biobank for one-sample MR

SNP	EA	NEA	EAF	Beta	SE	*P*-value
rs4949465	T	C	0.87	-0.017	0.003	1.70E-08
rs11210229	A	G	0.384	0.017	0.002	2.00E-16
rs71627581	G	A	0.889	0.019	0.003	1.60E-09
rs6935954	A	G	0.421	0.014	0.002	8.20E-12
rs1922018	C	T	0.364	0.014	0.002	3.00E-12
rs10226228	A	G	0.63	-0.016	0.002	2.00E-15
rs6962772	A	G	0.846	0.016	0.003	7.80E-09
rs2062882	G	A	0.587	-0.012	0.002	1.10E-08
rs2675638	G	A	0.581	0.012	0.002	1.30E-09
rs10879871	T	G	0.343	-0.014	0.002	5.00E-11
rs8614	C	A	0.817	-0.017	0.003	1.80E-10
rs76608582	C	A	0.953	0.031	0.005	3.20E-10
rs6119897	G	A	0.762	-0.018	0.002	3.60E-15
rs12481282	G	C	0.722	-0.013	0.002	7.80E-09

SNP, single-nucleotide polymorphisms; EA, effect allele; NEA, non-effect allele; EAF, effect allele frequency; SE, standard error

Table S2 Single-nucleotide polymorphisms and effect sizes for lifetime smoking index in UK Biobank for two-sample MR

SNP	EA	NEA	Beta	SE	P-value	F-statistics
rs10226228	A	G	-0.016	0.002	2.00E-15	64.00
rs1050847	C	T	0.011	0.002	1.40E-08	30.25
rs10879871	T	G	-0.014	0.002	5.00E-11	49.00
rs11210229	A	G	0.017	0.002	2.00E-16	72.25
rs11255908	T	G	-0.015	0.002	2.30E-10	56.25
rs11783093	C	T	0.023	0.003	1.20E-16	58.77
rs1193237	G	C	-0.011	0.002	2.80E-08	30.25
rs11948770	T	C	-0.015	0.002	4.90E-10	56.25
rs1221148	C	G	0.013	0.002	7.30E-11	42.25
rs12967855	A	G	0.012	0.002	3.10E-08	36.00
rs136233	A	G	-0.014	0.003	1.80E-08	21.77
rs17309874	G	A	-0.016	0.002	9.70E-13	64.00
rs17553262	A	C	-0.018	0.003	5.30E-09	36.00
rs17576594	G	A	0.016	0.002	1.70E-12	64.00
rs1922018	C	T	0.014	0.002	3.00E-12	49.00
rs2062882	G	A	-0.012	0.002	1.10E-08	36.00
rs2401924	G	C	0.015	0.002	2.70E-14	56.25
rs2675638	G	A	0.012	0.002	1.30E-09	36.00
rs2838834	C	T	-0.013	0.002	6.30E-10	42.25
rs2867112	T	G	0.021	0.003	4.80E-15	49.00
rs2894808	T	A	-0.022	0.004	3.50E-09	30.25
rs326341	G	A	0.014	0.002	1.20E-11	49.00
rs329120	C	T	0.014	0.002	6.30E-12	49.00
rs3742365	T	C	-0.016	0.002	2.50E-14	64.00
rs3769949	T	A	-0.012	0.002	2.50E-09	36.00
rs4391802	A	G	0.015	0.002	1.40E-11	56.25
rs4473348	A	T	-0.015	0.002	6.40E-11	56.25
rs4543592	T	C	-0.012	0.002	4.50E-10	36.00
rs4671357	T	C	-0.014	0.002	1.10E-11	49.00
rs4949465	T	C	-0.017	0.003	1.70E-08	32.11
rs4957528	A	C	-0.015	0.002	4.20E-09	56.25
rs6119897	G	A	-0.018	0.002	3.60E-15	81.00
rs624833	T	G	0.013	0.002	6.60E-10	42.25
rs6741228	T	C	0.011	0.002	1.60E-08	30.25
rs6778080	T	C	0.016	0.002	1.30E-12	64.00
rs6935954	A	G	0.014	0.002	8.20E-12	49.00
rs6962772	A	G	0.016	0.003	7.80E-09	28.44
rs7766610	C	A	0.018	0.003	2.20E-12	36.00
rs8614	C	A	-0.017	0.003	1.80E-10	32.11
rs889398	C	T	0.013	0.002	6.30E-11	42.25
rs986391	G	A	0.016	0.002	9.40E-15	64.00
rs9919670	G	A	-0.022	0.002	7.60E-27	121.00

SNP, single-nucleotide polymorphisms; EA, effect allele; NEA, non-effect allele; EAF, effect

allele frequency; SE, standard error. The summary-level statistics were extracted from a published genome-wide association studies in UK Biobank [37]

Table S3 Associations between the single-nucleotide polymorphisms included in the genetic instruments and potential confounders including diabetes, hypertension, and body mass index

SNP	Diabetes			Hypertension			Body mass index		
	Beta	SE	*P*-value	Beta	SE	*P*-value	Beta	SE	*P*-value
rs4949465	0.020	0.017	0.239	0.011	0.008	0.132	0.095	0.017	**3.04E-08**
rs11210229	-0.005	0.012	0.646	0.005	0.005	0.359	-0.007	0.012	0.539
rs71627581	0.020	0.017	0.239	0.015	0.007	0.051	0.077	0.017	6.17E-06
rs6935954	0.002	0.011	0.860	-0.005	0·005	0.366	0.008	0.012	0.510
rs1922018	0.012	0.012	0.322	-0.001	0.005	0.787	0.002	0.012	0.891
rs10226228	0.025	0.012	0.034	-0.004	0.005	0.455	0.055	0.012	3.71E-06
rs6962772	0.047	0.016	0.003	0.014	0.007	0.043	0.097	0.016	**8.20E-10**
rs2062882	-0.062	0.011	**4.73E-08**	-0.016	0.005	0.001	-0.073	0.012	**2.22E-10**
rs2675638	-0.003	0.011	0.817	-0.002	0.005	0.693	0.032	0.012	0.006
rs10879871	0.020	0.012	0.092	0.009	0.005	0.081	0.010	0.012	0.428
rs8614	-0.033	0.015	0.028	0.005	0.007	0.493	0.045	0.015	0.003
rs76608582	0.026	0.013	0.050	0.004	0.006	0.443	0.048	0.013	3e-4
rs6119897	0.009	0.013	0.492	0.016	0.006	0.007	0.020	0.014	0.149
rs12481282	-0.012	0.013	0.359	-0.007	0.006	0.206	0.003	0.013	0.825

SNP, single-nucleotide polymorphisms; SE, standard error. The association analyses were performed with the SNPs included in the genetic instrument for diabetes, hypertension and BMI by logistic regression analysis adjusted for age, sex, assessment center, and the first 10 principal components of the genetic information. SNPs that reached genome-wide significance ($P < 5\times10\text{-}8$, highlighted in red and bold) were excluded from the genetic instrument in the sensitivity analysis.

Table S5 Leave-one-out analysis results of one-sample mendelian randomization analysis

Excluded SNPs	OR (95% CI)	*p*-value
rs11210229	0.57(0.11,3.00)	0.51
rs71627581	0.59(0.12,2.98)	0.52
rs6935954	0.67(0.12,3.92)	0.66
rs1922018	0.81(0.12,5.51)	0.84
rs10226228	0.94(0.31,2.84)	0.91
rs2675638	0.82(0.15,4.46)	0.82
rs10879871	0.90(0.29,2.81)	0.85
rs8614	1.04(0.28,3.81)	0.96
rs76608582	0.01(0.00,15.62)	0.16
rs6119897	1.21(0.28,5.24)	0.80
rs12481282	0.96(0.26,3.55)	0.95

SNP, single-nucleotide polymorphism; OR, odds ratio; CI, confidence interval.

The one-sample mendelian randomization analysis was repeated by omitting each SNP from the polygenic risk score for exposure.

STROBE-MR checklist of recommended items to address in reports of Mendelian randomization studies[1,2]

Item No.	Section	Checklist item	Page No.	Relevant text from manuscript
1	**TITLE and ABSTRACT**	Indicate Mendelian randomization (MR) as the study's design in the title and/or the abstract if that is a main purpose of the study	1-2	Title, Abstract
	**INTRODUCTION**	s		
2	**Background**	Explain the scientific background and rationale for the reported study. What is the exposure? Is a potential causal relationship between exposure and outcome plausible? Justify why MR is a helpful method to address the study question	3-4	Introduction: paragraph 1-3
3	**Objectives**	State specific objectives clearly, including pre-specified causal hypotheses (if any). State that MR is a method that, under specific assumptions, intends to estimate causal effects	3-4	Introduction: paragraph 3
	**METHODS**			
4	**Study design and data sources**	Present key elements of the study design early in the article. Consider including a table listing sources of data for all phases of the study. For each data source contributing to the analysis, describe the following:		
	a)	Setting: Describe the study design and the underlying population, if possible. Describe the setting, locations, and relevant dates, including periods of recruitment, exposure, follow-up, and data collection, when available.	4	Methods: Method overview, Study population,
	b)	Participants: Give the eligibility criteria, and the sources and methods of selection of participants. Report the sample size, and whether any power or sample size calculations were carried out prior to the main analysis	4	Methods: Study population, Supplementary Material: Supplementary Methods
	c)	Describe measurement, quality control and selection of genetic variants	5-6	Methods: Genetic instruments
	d)	For each exposure, outcome, and other relevant variables, describe methods of assessment and diagnostic criteria for diseases	4-5	Methods: Study population, Smoking index Supplementary Material: Supplementary Methods
	e)	Provide details of ethics committee approval and participant informed consent, if relevant	4,15	Methods: Study population, Ethical Approval
5	**Assumptions**	Explicitly state the three core IV assumptions for the main analysis (relevance, independence and exclusion restriction) as well assumptions for any additional or sensitivity analysis	13-14	Discussion: paragraph 5

6	**Statistical methods: main analysis**		Describe statistical methods and statistics used		
		a)	Describe how quantitative variables were handled in the analyses (i.e., scale, units, model)	5-9	Methods: Genetic instruments, Statistical Methods: Traditional observational study, One-sample MR, Two-sample MR
		b)	Describe how genetic variants were handled in the analyses and, if applicable, how their weights were selected		
		c)	Describe the MR estimator (e.g. two-stage least squares, Wald ratio) and related statistics. Detail the included covariates and, in case of two-sample MR, whether the same covariate set was used for adjustment in the two samples		
		d)	Explain how missing data were addressed		
		e)	If applicable, indicate how multiple testing was addressed		
7	**Assessment of assumptions**		Describe any methods or prior knowledge used to assess the assumptions or justify their validity	5-9	Methods: Genetic instruments, Statistical Methods: One-sample MR: paragraph 2, Two-sample MR: paragraph 1-2
8	**Sensitivity analyses and additional analyses**		Describe any sensitivity analyses or additional analyses performed (e.g. comparison of effect estimates from different approaches, independent replication, bias analytic techniques, validation of instruments, simulations)	7-9	Methods: Statistical Methods: One-sample MR: paragraph 2, Two-sample MR: paragraph 2
9	**Software and pre-registration**				
		a)	Name statistical software and package(s), including version and settings used	8-9	Methods: Statistical Methods: One-sample MR, Two-sample MR
		b)	State whether the study protocol and details were pre-registered (as well as when and where)		N/A
	**RESULTS**				
10	**Descriptive data**				
		a)	Report the numbers of individuals at each stage of included studies and reasons for exclusion. Consider use of a flow diagram	9-10	Results: Study population characteristics in UKB, Figure 1

		b)	Report summary statistics for phenotypic exposure(s), outcome(s), and other relevant variables (e.g. means, SDs, proportions)	9	Results: Study population characteristics in UKB, Table 1
		c)	If the data sources include meta-analyses of previous studies, provide the assessments of heterogeneity across these studies		N/A
		d)	For two-sample MR:<br>i. Provide justification of the similarity of the genetic variant-exposure associations between the exposure and outcome samples<br>ii. Provide information on the number of individuals who overlap between the exposure and outcome studies	11	Results: Two-sample MR analysis: paragraph 1
11	**Main results**				
		a)	Report the associations between genetic variant and exposure, and between genetic variant and outcome, preferably on an interpretable scale	10-11	Results: One-sample MR analysis: paragraph 1, Table S1, Two-sample MR analysis: paragraph 1 Table S2
		b)	Report MR estimates of the relationship between exposure and outcome, and the measures of uncertainty from the MR analysis, on an interpretable scale, such as odds ratio or relative risk per SD difference	10-11	Results: One-sample MR analysis: paragraph 1, Table 3, Two-sample MR analysis: paragraph 1 Table 4
		c)	If relevant, consider translating estimates of relative risk into absolute risk for a meaningful time period		N/A
		d)	Consider plots to visualize results (e.g. forest plot, scatterplot of associations between genetic variants and outcome versus between genetic variants and exposure)	11	Results: Two-sample MR analysis: Figure 3
12	**Assessment of assumptions**				
		a)	Report the assessment of the validity of the assumptions	10-11	Results: One-sample MR analysis: paragraph 1-2, Two-sample MR analysis, paragraph 1, Table S1-S5
		b)	Report any additional statistics (e.g., assessments of heterogeneity across genetic variants, such as I^2, Q statistic or E-value)		
13	**Sensitivity analyses and additional analyses**				

		a)	Report any sensitivity analyses to assess the robustness of the main results to violations of the assumptions	10-11	Results: One-sample MR analysis: paragraph 2, Two-sample MR analysis, Table S3-S5, Figure S1
		b)	Report results from other sensitivity analyses or additional analyses		
		c)	Report any assessment of direction of causal relationship (e.g., bidirectional MR)		
		d)	When relevant, report and compare with estimates from non-MR analyses	10-11	Results: Traditional observational study
		e)	Consider additional plots to visualize results (e.g., leave-one-out analyses)	11	Results: Two-sample MR analysis, Figure 3
	**DISCUSSION**				
14	**Key results**		Summarize key results with reference to study objectives	11-12	Discussion: paragraph 1-3
15	**Limitations**		Discuss limitations of the study, taking into account the validity of the IV assumptions, other sources of potential bias, and imprecision. Discuss both direction and magnitude of any potential bias and any efforts to address them	13-14	Discussion: paragraph 5,6
16	**Interpretation**				
		a)	Meaning: Give a cautious overall interpretation of results in the context of their limitations and in comparison with other studies	12-13	Discussion: paragraph 3,4
		b)	Mechanism: Discuss underlying biological mechanisms that could drive a potential causal relationship between the investigated exposure and the outcome, and whether the gene-environment equivalence assumption is reasonable. Use causal language carefully, clarifying that IV estimates may provide causal effects only under certain assumptions		
		c)	Clinical relevance: Discuss whether the results have clinical or public policy relevance, and to what extent they inform effect sizes of possible interventions		
17	**Generalizability**		Discuss the generalizability of the study results (a) to other populations, (b) across other exposure periods/timings, and (c) across other levels of exposure	14	Discussion: paragraph 6
	**OTHER INFORMATION**				
18	**Funding**		Describe sources of funding and the role of funders in the present study and, if applicable, sources of funding for the databases and original study or studies on which the present study is	15	Funding

		based		
19	**Data and data sharing**	Provide the data used to perform all analyses or report where and how the data can be accessed, and reference these sources in the article. Provide the statistical code needed to reproduce the results in the article, or report whether the code is publicly accessible and if so, where	15	Data Availability
20	**Conflicts of Interest**	All authors should declare all potential conflicts of interest	16	Conflict of interest

This checklist is copyrighted by the Equator Network under the Creative Commons Attribution 3.0 Unported (CC BY 3.0) license.

1. Skrivankova VW, Richmond RC, Woolf BAR, Yarmolinsky J, Davies NM, Swanson SA, et al. Strengthening the Reporting of Observational Studies in Epidemiology using Mendelian Randomization (STROBE-MR) Statement. JAMA. 2021;under review.

2. Skrivankova VW, Richmond RC, Woolf BAR, Davies NM, Swanson SA, VanderWeele TJ, et al. Strengthening the Reporting of Observational Studies in Epidemiology using Mendelian Randomisation (STROBE-MR): Explanation and Elaboration. BMJ. 2021;375:n2233.
