# Supplementary figures and images for: Association of Smoking with Chronic Kidney Disease Stages 3 to 5: A Mendelian Randomization Study
Source: Health Data Sci. 2024 Nov 4;4:0199. doi: 10.34133/hds.0199 (PMC11532587; doi:10.34133/hds.0199)

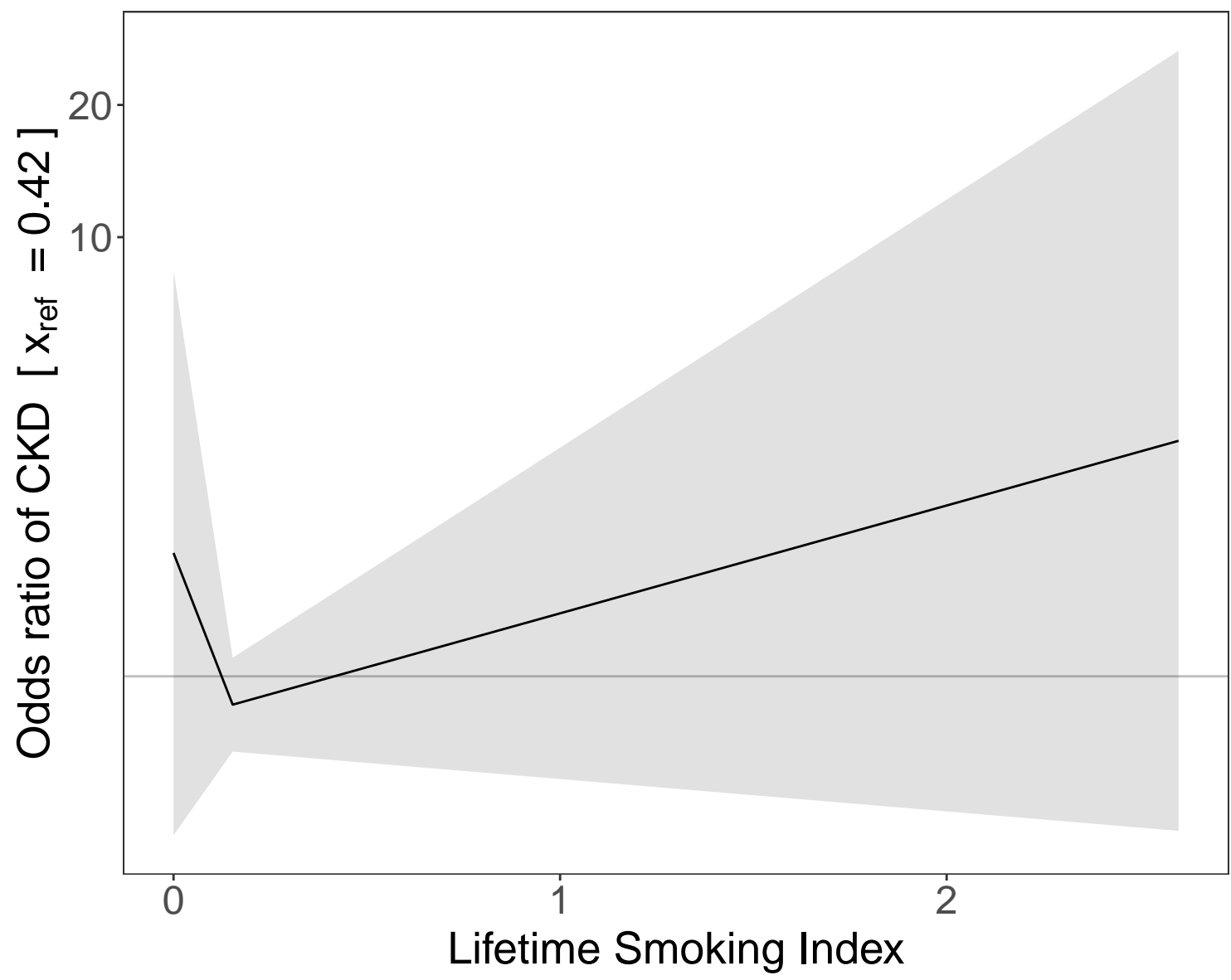

Supplement: Supplementary 1 — Supplementary Methods Supplementary Results Fig. S1 Tables S1 to S5 References [69,70] [file hds.0199.f1.zip › FigureS1_nonlinear.pdf]
